# Ecological risk and protective factors for food insufficiency in Los Angeles County during the COVID-19 pandemic

**DOI:** 10.1017/S1368980023001337

**Published:** 2023-10

**Authors:** Kayla de la Haye, Htay-Wah Saw, Sydney Miller, Wändi Bruine de Bruin, John P Wilson, Kate Weber, Alison Frazzini, Michelle Livings, Marianna Babboni, Arie Kapteyn

**Affiliations:** 1 Department of Population and Public Health Sciences, Keck School of Medicine, University of Southern California, Los Angeles, CA, USA; 2 Center for Economic and Social Research, Dornsife College of Letters Arts and Sciences, University of Southern California, Los Angeles, CA, USA; 3 Institute for Social Research, Survey Research Center, University of Michigan-Ann Arbor, Ann Arbor, MI, USA; 4 Dornsife Department of Psychology, University of Southern California, Los Angeles, CA, USA; 5 Schaeffer Center for Health Policy and Economics, Price School of Public Policy, University of Southern California, Los Angeles, CA, USA; 6 Spatial Sciences Institute and Department of Sociology, Dornsife College of Letters, Arts, and Sciences, University of Southern California, Los Angeles, CA, USA; 7 Civil and Environmental Engineering and Computer Science, Viterbi School of Engineering, University of Southern California, Los Angeles, CA, USA; 8 School of Architecture, University of Southern California, Los Angeles, CA, USA; 9 USC Dornsife Public Exchange, University of Southern California, Los Angeles, CA, USA; 10 Chief Sustainability Office, County of Los Angeles, Los Angeles, CA, USA

**Keywords:** Food insufficiency, Food insecurity, COVID-19, Los Angeles County, Food assistance, Supplemental Nutrition Assistance Program

## Abstract

**Objective:**

The COVID-19 pandemic increased food insufficiency: a severe form of food insecurity. Drawing on an ecological framework, we aimed to understand factors that contributed to changes in food insufficiency from April to December 2020, in a large urban population hard hit by the pandemic.

**Design:**

We conducted internet surveys every 2 weeks in April–December 2020, including a subset of items from the Food Insecurity Experience Scale. Longitudinal analysis identified predictors of food insufficiency, using fixed effects models.

**Setting:**

Los Angeles County, which has a diverse population of 10 million residents.

**Participants:**

A representative sample of 1535 adults in Los Angeles County who are participants in the Understanding Coronavirus in America tracking survey.

**Results:**

Rates of food insufficiency spiked in the first year of the pandemic, especially among participants living in poverty, in middle adulthood and with larger households. Government food assistance from the Supplemental Nutrition Assistance Program was significantly associated with reduced food insufficiency over time, while other forms of assistance such as help from family and friends or stimulus funds were not.

**Conclusions:**

The findings highlight that during a crisis, there is value in rapidly monitoring food insufficiency and investing in government food benefits.

The COVID-19 pandemic caused a global surge in food insecurity and food insufficiency, including in the USA among some vulnerable populations (e.g. low income, unemployed) and in some regions hard hit by the pandemic^(1–5)^. *Food insecurity* refers to the disruption of food intake or eating patterns because of lack of money and other resources, and historically it is experienced by one in ten U.S. households annually^(6)^. *Food insufficiency* is a more severe dimension of food insecurity where households do not have enough to eat^(7)^.

National and state-level statistics from U.S.D.A. surveys found that 10·5 % of U.S. residents reported food insecurity in the past year, at the end of 2019 and the end of 2020, with the rate remaining stable at 10·5 % both years^(8)^. However, these annual surveys may not have accurately captured people’s experiences during the first months of the pandemic^(9)^. Survey data from the U.S. Census Bureau collected regularly throughout the pandemic suggest that rates of food insufficiency did increase in the first months of the pandemic^(3)^. Local studies also documented a rise in food insecurity, especially among some high risk populations such as low-income African Americans^(10)^. Issues of food access, and its causes and solutions^(11)^, may be unique during the pandemic due to the complex and sustained disruptions to economic systems (e.g. job loss), social systems (e.g. social distancing, closure of public institutions) and food systems (e.g. closure and limited hours and capacity of food outlets, changing landscape of food assistance).

Food insecurity, which we use in this literature review to be inclusive of the experience of food insufficiency, is associated with negative health outcomes for children and adults, including poor nutrition, mental health problems and increased risk for diet-related diseases like obesity and hypertension^(11,12)^. Although economic hardships are a primary cause of these food issues, the risk factors are complex and include having fewer assets (e.g. owning a home), high cost of living and food prices, single parent households and less education^(11)^. Food insecurity can be triggered by disruptions in household members’ income, employment and health^(11)^. It may also be exacerbated by shocks to broader ecological systems^(13)^.

There is evidence that food insecurity can be alleviated through formal and informal food assistance. Government-administered food programmes, the largest in the USA being the Supplemental Nutrition Assistance Program (SNAP), provide low-income households with money to spend on food^(14)^. In 2019, SNAP provided 38 million Americans with an average $250/month^(15)^ and has been shown to ameliorate food security^(11,16,17)^. Additionally, emergency assistance from charitable food assistance programmes, such as food pantries, provided free food to more than 46 million Americans annually prior to the pandemic^(18)^. Although emergency food assistance programmes were primarily established to relieve acute food needs, they are used as a supplement by recipients of governmental food assistance and as a primary source of food assistance by people without governmental food assistance^(19)^. Emergency food assistance can have positive short-term benefits to nutrition and food security^(19,20)^. More broadly, people’s social networks – their connections to family, friends, neighbours and community organisations – can also serve as a buffer against food insecurity through the provision of food assistance and social capital^(21,22)^.

## Food insecurity in the context of the COVID-19 pandemic

Few studies have regularly monitored food insecurity during sustained crises or comprehensively assessed the associated risks and solutions as these dynamics unfolded within a changing system. Most research on food insecurity in the context of the COVID-19 pandemic has been cross-sectional in nature. Cross-sectional research has identified factors associated with a higher risk for food insecurity and food insufficiency among U.S. adults during the pandemic, including being on low income, belonging to race and ethnic minorities, and having children in the household^(23)^, as well as differences in risk across geographic areas (e.g. urban *v*. rural, and metropolitan regions within a state)^(24,25)^. One drawback of cross-sectional studies is that the temporal direction of findings remains unclear. For example, positive relationships at any one point in time between food insecurity and assistance programmes (e.g. SNAP) may mean that food assistance programmes are not working, but they may also be due to people experiencing food insecurity being more likely to *seek out* these programmes^(26)^. Longitudinal data are needed to examine if programme use predicts transitions from experiencing food insecurity to being food secure.

However, longitudinal research that comprehensively examines ecological risk and protective factors that may contribute to the onset and relief of food insecurity is lacking. One of the few studies that did report longitudinal analyses found that U.S. adults who became unemployed during the COVID-19 pandemic experienced increases in food insufficiency and decreases in food expenditures and confidence in the ability to afford food^(27)^. Another study on this population found that food insecurity was highest at the beginning of the pandemic (22 % in April 2020) and subsequently declined^(28)^, and that receiving unemployment insurance decreased food insecurity^(28)^.

## The present study

**Fig. 1:**
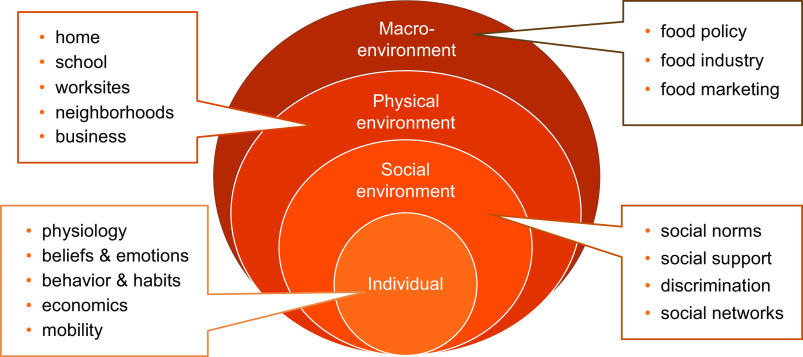
Ecological influences on food access, diet and food security

Here, we conducted a comprehensive longitudinal study in Los Angeles (L.A.) County, the most populous U.S. county whose 10 million economically and ethnically diverse residents were hard hit by the pandemic. We draw on a social ecological model of health promotion^(29,30)^ that highlights the need for identifying individual factors as well as factors in the social, physical, and macro environment that influence food access and diet^(31,32)^ (Fig. 1). Because the pandemic’s disruptions have altered many of these factors, we examine multilevel risk and protective factors that may be associated with food insufficiency or not having enough to eat. For example, job losses in L.A. County increased unemployment from 5 % in February 2020 to 19 % in May 2020^(33)^. This coincided with constraints in the ‘last mile’ of food distribution as restaurants closed, and people lost access to meals at schools and community centres. Many residents were also were cut off from their social networks and the food support they provide^(21)^. Government and community organisations responded by expanding existing food assistance programmes and creating new ones. For example, the federal government established the Pandemic Electronic Benefit Transfer (Pandemic EBT) food assistance programme that provided additional benefits to spend on food, to families with children who were eligible for free or reduced-price school meals^(34)^. The landscape of charitable food assistance programmes also changed, with some organisations closing, others expanding capacity and new food pantries ‘popping-up’ to meet growing needs. In this changing and complex environment, it is not obvious what key risk factors for food adequacy have emerged (or persisted), and what solutions are working.

This longitudinal study was conducted from April to December 2020, with a large and representative sample of L.A. County households (*n* 1535)^(35)^. Building on the social ecological framework outlined above^(29,30)^ (Fig. 1), we examined multilevel risk factors for food insufficiency and expected that poverty, as well as other personal, social and environmental barriers to food access would exacerbate this risk. We also examined the role of established and new food assistance programmes in reducing food insufficiency.

## Methods

### Study procedure and participants

Data come from the Understanding Coronavirus in America tracking survey, augmented by contextual data on food environments from the U.S.D.A. Food Access Research Atlas^(36)^. Members of the Understanding America Study (UAS) consented to participate in the Understanding Coronavirus in America tracking survey. The UAS is a probability-based nationally representative internet panel of U.S. individuals 18 and older, and the Understanding Coronavirus in America component surveyed households throughout the pandemic. To obtain a representative sample, UAS participants were recruited from randomly selected U.S. addresses. Sampling probabilities were adjusted for underrepresented populations, and internet-connected tablets were provided to interested individuals if needed. UAS protocols, including those relevant to this study, were approved by the University of Southern California Institutional Review Board.

Surveys to assess food insufficiency, as well as risk and protective factors identified by the social ecological framework^(30)^, were fielded with a sub-sample of Understanding Coronavirus in America participants representative of L.A. County. Therefore this study focused exclusively on participants in L.A. County (*n* 1535), and their survey responses across nineteen waves conducted from 1 April to 23 December 2020. Participants were invited to respond to survey interviews every 2 weeks and were assigned a fixed day in the 2-week cycle of each survey wave (e.g. the second Wednesday in the 2-week period). They were given 2 weeks to complete the survey, until their invite for the next survey wave. Since the respondents on the last day of the 2-week cycle had 2 weeks to complete their survey, the final responses for every survey cycle (i.e. wave) were collected at most 4 weeks after the start of a wave. The majority (81 %) of participants responded on their assigned day, 96 % were recorded within the first 2 weeks of the survey wave and the remaining 4 % were completed by the end of the 4-week wave. The average annual attrition in the L.A. County sub-sample of the UAS is about 13 % annually, inclusive of the data collected from this sample in 2020 via the Understanding Coronavirus in America tracking survey. Post-stratification weights were used to further align the L.A. sample to L.A. County’s population regarding age, gender, race/ethnicity and education^(37)^.

### Measures

Food insufficiency was measured every wave using a subset of three items from the validated Food Insecurity Experience Survey (FIES)^(38)^, which was selected because of its brevity and validity^(39)^. Of the eight FIES items, we selected three with the highest factor loadings for each level of food insecurity: mild (*‘In the past 7 days, were you worried you would run out of food because of a lack of money or other resources?’*), moderate (*‘In the past 7 days, did you eat less than you thought you should because of a lack of money or other resources?’*) and severe (*‘In the past 7 days, did you go without eating for a whole day because of a lack of money or other resources’*) (Table 1). As is standard in the literature, participants were treated as expressing food insufficiency if they gave a positive response to either or both of the questions about moderate or severe food insecurity in the past week (Table 1)^(7,38)^. We use the label ‘food insufficient’ in this study, instead of ‘food insecure’, because we have used a subset of items from the FIES that capture the more narrow and severe experience of ‘not having enough to eat’. All other participants, including those who indicated worry about food without indicating moderate or severe food insecurity (Table 1), were categorised as ‘not food insufficient’. We note that in a study using the U.S.D.A. Household Food Security Module, assessments of food insecurity were found to be highly correlated (*r* > 0·9) independent of whether they had 1, 6, 10 or 18 items; only the single-item measure was found to lead to underestimation^(40)^. In this study, the correlation coefficient for the two items used to assess food insufficiency was *r* = 0·50 (*p* <.01).

**Table 1:** Food insufficiency measure

Food Insecurity Experiences Scale (FIES) items In the past 7 days, […] because of a lack of money or other resources?	Level	Included in measure of food insufficiency
*…did you go without eating for a whole day…*	Severe	X
*…did you eat less than you thought you should…*	Moderate	X
*…were you worried you would run out of food…*	Mild	

#### Explanatory variables

##### Demographics and health

Demographics, reported every 3 months, included: (i) gender (male, female); (ii) age in years (categorised as 18–30, 31–40, 41–50, 51–64 and 65+), (iii) race and ethnicity (categorised as Hispanic/Latinx, White (non-Hispanic), Black (non-Hispanic), Asian (non-Hispanic), American Indian/Alaskan Native, Hawaiian/Pacific Islander and Other); (iv) education level (categorised as having a high school degree, GED or equivalent, or less; or some college or college and above); (v) employment status (used to create two binary variables: unemployed and unemployed because of disability) and (vi) annual household income and household size were used to compute the percentage of the Federal Poverty Level (FPL) and then create two binary variables: ‘living in poverty (< 100 % FPL)’ and ‘low income (< 300 % FPL)’^(41)^. A diagnosis of COVID-19 was assessed at each survey wave with the question *‘Whether or not you have had a coronavirus test, has a doctor or another healthcare professional diagnosed you as having or probably having the coronavirus since (the date of previous survey)?*’.

##### Household and social characteristics

As part of standard UAS procedures, the number of adults and children in the household is updated every 3 months. The most recent report of these measures was used to identify the following variables used in this study: household size, any children in the household and single parent households with children. Social network size and support have been associated with food insecurity^(21)^. Social circle size was assessed monthly with the question ‘*About how many friends and family members do you have*?’ (followed by a request to confirm the number was correct), and this number was log transformed for analyses. Social support to access food was assessed in July with the question ‘*In the past 30 days, how many of these family and friends helped you to get enough food to eat, by sharing money, resources, or food with you*?’. The percentage of one’s social circle that helped provide food was calculated by dividing the number that provided food support by the social circle size.

##### Food access and food environment

Participants were coded as living in a low food access neighbourhood, with limited spatial access to a grocery store (also known as a ‘food desert’), if they lived in a census tract defined as ‘low access tract at 1 mile for urban areas or 10 miles for rural areas’ in the U.S.D.A. Food Access Research Atlas^(36)^. (We also explored U.S.D.A. indicators for census tracts that are both ‘low access’ and ‘low income’, but this did not change the results).

Because access to a personal vehicle has been associated with food access and a significantly lower risk of food insecurity^(42)^ in the past, we included two measures related to transportation. Every 3 months, respondents were asked ‘*How many private vehicles (cars, vans, trucks, or SUVs) in working condition does your household currently own or lease*?’, and their most recent response was used to identify participants with no (0) vehicle. In July, respondents were asked if they had challenges getting food because of lack of car or personal transportation (Yes/No).

##### Receipt of programmes and benefits

In all survey waves except 8–21 July, participants indicated whether anyone in their household had received any of the following government benefits in the past month (Yes/No): SNAP or Food Stamps; Special Supplemental Nutrition Program for Women, Infants and Children; unemployment insurance; Social Security; Supplemental Security Income; Social Security Disability Insurance; economic stimulus funds; aid for people or businesses affected by the coronavirus epidemic. Receipt of these benefits at each survey wave was treated as a time-varying explanatory variable. Receipt of Pandemic EBT benefit of up to $136/month for families whose children are eligible for funded school meals (described as ‘Pandemic EBT benefits for children in your household, to help pay for food because schools are closed’) was assessed at the end of the year (January 2021). At one survey wave (April 29–May 12), participants indicated whether they had received food from a food bank or food pantry in the past two weeks (Yes/No).

We also explored if the amount of money people received from these programmes predicted food insufficiency. However, these variables were excluded from the final models because they did not add predictive value and were not assessed at all waves or for all types of benefits.

### Statistical analysis

Variables were checked for accuracy of responses, particularly with regard to the receipt of benefits. For example, if participants younger than 62 years reported receiving Social Security benefits, we assumed they were not likely to be eligible and this was corrected. After the data cleaning, the results of the statistical analysis were essentially unchanged.

We computed descriptive statistics for all variables used in the analyses. Longitudinal analysis to identify predictors of food insufficiency was performed using fixed effects models^(43)^. A challenge for the analyses we have undertaken is that not all characteristics of respondents are captured by the data. For example, raw correlations between food insufficiency and receipt of SNAP benefits are positive, which could be interpreted as the receipt of SNAP benefits increasing someone’s risk for food insufficiency. A more plausible interpretation is that those who receive SNAP are generally in need of food assistance, a characteristic that is not fully captured by our data – although poverty level is one imperfect proxy. For this reason, we employed fixed effects models to account for non-time-varying individual factors that are not captured by our data.

The fixed effects model employed takes into account individual fixed effects but exploits a result derived by Mundlak^(43)^, which shows that the estimates of time-varying explanatory variables are identical to a procedure where one does not include fixed effects but rather includes the mean of all time-varying variables as additional regressors. The coefficients of these means are interpreted as estimates of the correlation of the unobserved individual effects with the time-varying variables. The advantage of this procedure is that one can retain all non-time-varying explanatory variables and estimate their effects on the outcomes of interest. Thus, the advantage of the use of a fixed effects models is that it accounts for the effect of non-observed time invariant factors and in that way accounts for possible reverse causality. For example, as in the example above where the positive correlation between food insufficiency and SNAP receipt plausibly reflects unobserved factors that make families at higher risk of food insufficiency more likely to be SNAP recipients. The general interpretation of the estimated coefficients of time-varying explanatory variables (like SNAP receipt or employment status) is that they measure the effect of *changes* in the explanatory variables on *changes* in the dependent variable (food insufficiency in this case). Since we consider brief 2-week periods, these estimates reflect short-term effects (e.g. how SNAP receipt within a 2-week period affects food insufficiency in that period). The effects of non-time-varying variables are by construction constant during the observation period. For example, the effect of education on food insufficiency is assumed not to vary during the sample period. An additional benefit of using fixed effects models is that they account for the correlation in longitudinal data from the same individual, which may be due to unobserved factors that differ between individuals such as personality and survey response styles. Fixed effects models eliminate these sources of correlation between waves.

We also fit ordinary least squares regression models, which do not take into account unobserved individual characteristics, to explore the stability of results across the two approaches. The results presented are consistent with the trends identified in the ordinary least squares. Survey weights were not included in the regressions or fixed effects models as this would amount to assuming heteroskedasticity (i.e. the error term has different variances for different observations), where respondents with high weights would be assumed to give the most accurate information. There is no reason for such an assumption, and so weighting would likely make the statistical inference less precise. The results of the fixed effects model and significant effects (*P* < 0·05) are described in the results.

## Results

### Descriptive statistics

**Fig. 2:**
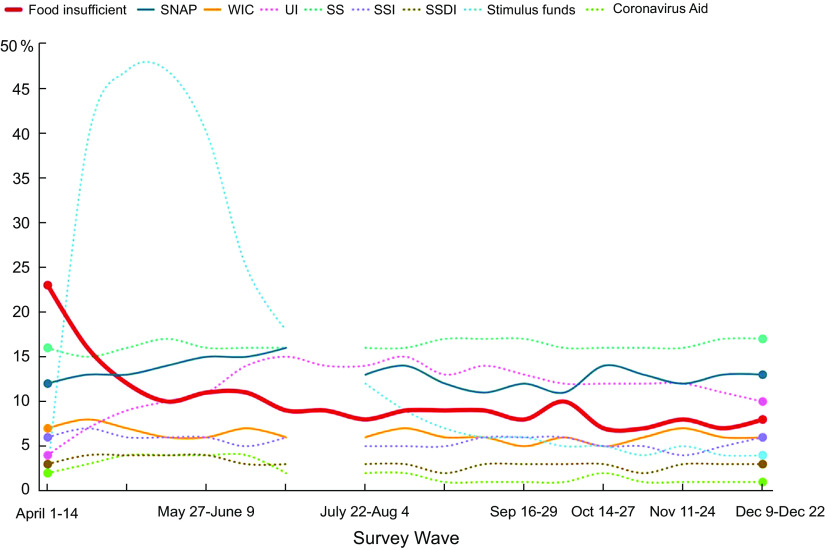
Percent of the L.A. County adult population that experienced past week food insufficiency and received government benefits, by survey wave (weighted statistics). SNAP = Supplemental Nutrition Assistance Program; WIC = Special Supplemental Nutrition Program for Women, Infants and Children; UI = Unemployment Insurance; SS = Social Security, SSI = Supplemental Security Income; SSDI = Social Security Disability Insurance. *Note.* Most benefits were not assessed in UAS 252, 8–21 July

Between April and December 2020, the average rate of *past week* food insufficiency across all surveys was 10 % (Table 2). However, rates of past week food insufficiency differed markedly over time, peaking at 23 % during the beginning of April, when our survey started just weeks after L.A. County ‘stay at home’ orders were issued, declining to 12 % by early May, and fluctuating between 8 and 11 % in June–December (Fig. 2). Among respondents who completed all nineteen survey waves, we found that 26 % had experienced food insufficiency *at one or more* waves from April to December 2020.

**Table 2.** Descriptive statistics (unweighted) for food insufficiency and explanatory variables, split by adults who were food sufficient *v*. food insufficient (at any time) between April and December 2020

		All (*n* 1535)			Food sufficient (*n* 1214)			Food insufficient (*n* 297)		
Variable	Obs.	%	Mean	sd	%	Mean	sd	%	Mean	sd
Food insufficiency in the past week	21 295	10·1								
Demographics and health										
Male	1535	47·1			49·8			39·0		
Age										
18–30 years	1532	22·7			18·71			34·4		
31–40 years	1532	23·2			22·4			26·5		
41–50 years	1532	16·4			16·6			16·7		
51–64 years	1532	20·8			22·4			15·8		
65+ years	1532	16·9			19·9			6·5		
Race and ethnicity										
Hispanic/Latinx (White)	1494	45·4			42·9			54·0		
White (non-Hispanic)	1494	24·8			27·8			14·7		
Black (non-Hispanic)	1494	7·7			7·0			9·9		
Asian (non-Hispanic)	1494	15·0			15·9			12·5		
All American Indian/Alaskan Native	1494	1·0			1·0			0·1		
All Hawaiian/Pacific Islander	1494	0·5			0·5			0·3		
All Others	1494	5·6			4·9			7·7		
Education										
GED or equivalent, or less	1535	45·0			40·2			60·5		
Some college	1535	23·3			23·8			22·1		
College and above	1535	31·7			36·0			17·4		
Household income										
Living in poverty (< 100 % FPL)	1444	21·0			15·7			40·5		
Low income (< 300 % FPL)	1444	59·8			53·1			83·0		
Unemployed	21 710	22·2			20·3			38·1		
Not employed because of disability	21 704	6·4			6·1			9·2		
Diagnosed with coronavirus	21 614	0·5			0·4			1·0		
Household and social factors										
Household size	1446		2·88	1·57		2·83	1·51		3·01	1·76
Have children in the household	1535	32·5			30·8			36·5		
Single parent with children	1535	4·1			2·9			8·6		
Social circle size*	1465		26·40	41·23		27·74	42·43		21·37	36·56
Log social circle size	1449		2·68	1·07		2·74	1·06		2·46	1·09
Food access and environment										
Low food access neighbourhood†	1446	11·7			12·6			9·0		
Household does not have a vehicle‡	1268	8·7			8·0			12·0		
Difficulty getting food because no car§	1167	7·4			3·8			20·7		
Receipt of support, programmes and benefits										
% of social circle that helped with food||	1140		0·11	0·25		0·10	0·25		0·14	0·27
Received food from a food bank/pantry	1198	4·9			4·3			7·0		
SNAP	20 095	13·0			11·8			23·6		
WIC	20 084	6·3			5·9			9·4		
Pandemic EBT	1051	18·2			15·5			24·5		
Unemployed × unemployment insurance	21 381	5·8			5·8			6·4		
Social security	20 088	16·6			17·8			6·5		
Supplemental Security Income	20 091	5·7			5·4			8·0		
Social Security Disability Insurance	20 090	3·1			2·9			5·0		
Economic stimulus funds	20 095	16·3			15·8			20·4		
Aid for people affected by the coronavirus	20 099	2·1			2·1			1·9		

Obs., number of observations over all waves; GED, Tests of General Educational Development; FPL, Federal Poverty Level; SNAP, Supplemental Nutrition Assistance Program; WIC, Special Supplemental Nutrition Program for Women, Infants, and Children.

For variables that are assessed at multiple waves (time varying), the data from all waves were used to compute the summary statistics, while for variables assessed at only one wave (time invarying), data from that one observation were used to compute the summary statistics. For example, variables assessed at all nineteen waves for all 1535 participants have 27 630 potential observations, while variables assessed at one wave for all 1535 participants have 1535 potential observations. Thus, the variables are represented as they are analysed in the statistical models.

* Social circle size = the number of family and friends they have.

† Low food access neighbourhood = a census tract defined as a food desert based on the U.S.D.A.’s definition of ‘low access tract at 1 mile for urban areas or 10 miles for rural areas’.

‡ Household does not have a vehicle = participant reported that their household does not own or lease any private vehicles.

§ Difficulty getting food because no car = participant reported that they had challenges getting food because they lacked a car or personal transportation.

|| % of social circle that helped with food = participant’s estimation of the proportion of their family and friends (social circle) that helped them to get enough food to eat, by sharing money, resources or food.

Table 2 presents descriptive statistics for all variables included in the analysis for all participants and split by participants who experienced food insufficiency (i) never and (ii) at any time between April and December. The descriptive statistics show a higher rate of food insufficiency among women, younger adults, Hispanic/Latinx and people reporting less education, low incomes, unemployment and lacking personal transportation. Rates of food insufficiency also varied with receiving food assistance from family and friends, food pantries and government programmes, including SNAP, Special Supplemental Nutrition Program for Women, Infants and Children and the new Pandemic EBT benefit. Some challenges with food access were more common among people experiencing food insufficiency (Table 2). The proportion of people living in low food access neighbourhoods was higher among people who were food sufficient (13 %) *v*. food insufficient (9 %).

Figure 2 and the associated online supplementary material, Supplemental Table 1 present weighted descriptive statistics for rates of food insufficiency and the time-varying explanatory variables over the nineteen survey waves. They show that the percentage of the L.A. County population receiving many government benefits was stable, while SNAP use increased from 12 % in April to 16 % in July, an increase that is consistent with local government records^(44)^. Most households that received government economic stimulus funds and ‘coronavirus aid’ did so between April and July.

### Predictors of food insufficiency

**Fig. 3:**
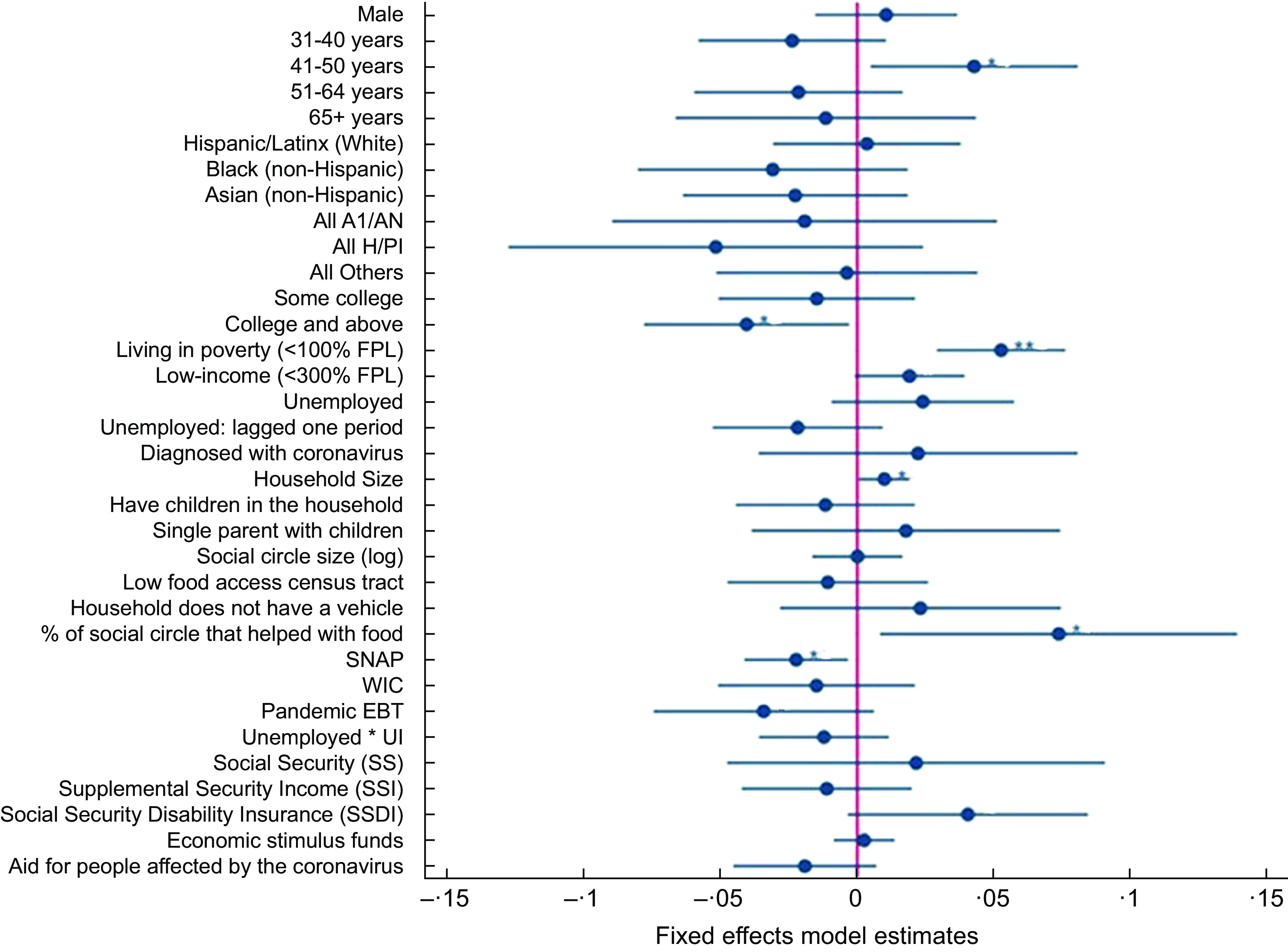
Fixed effects model results: Predictors of past week food insufficiency in L.A. County, April to December 2020. Points represent the estimate for each variable, and lines the 95% confidence interval around the estimate. AI/AN = American Indian/Alaskan Native; H/PI = Hawaiian/Pacific Islander; FPL = Federal Poverty Level; SNAP = Supplemental Nutrition Assistance Program; WIC = Special Supplemental Nutrition Program for Women, Infants, and Children; UI = Unemployment insurance *P < 0·05, **P < 0·01, ***P < 0·001

Figure 3 (online supplementary material, Supplemental Table 2) shows that living in poverty predicted food insufficiency (est. = 0·05, *P* < 0·001). Given the average 10 % rate of food insufficiency, having a household income < 100 % FPL increased the risk of food insufficiency by 53 % (i.e. 5·3 percentage points on a base of 10 %). Age also predicted food insufficiency: relative to 18–30-year-olds, the risk for food insufficiency was lower for all age groups *except* 41–50-year-olds. Being 41–50 years old increased the risk for food insufficiency by 43 % (est. = 0·04, *P* = 0·03). Finally, having a larger household significantly increased risk for food insufficiency (est. = 0·01, *P* = 0·04), with each additional household member increasing the risk by 10 %. Having a college education significantly *lowered* risk for food insufficiency by 41 % (est. = –0·04, *P* = 0·04). Other demographic, health and household factors did not significantly predict food insufficiency

Challenges with food access did not predict food insufficiency. The null effect of living in a low food access neighbourhood (est. = –0·01, *P* = 0·56) may partly be explained by some wealthy areas in L.A. County having few grocery stores, although additional analyses indicated living in a *low-income* food desert also did not predict food insufficiency. Although 12 % of people who experienced food insufficiency did not have a vehicle (*v*. 8 % of those who were food sufficient), and 21 % reported having difficulty getting food because they did not have a car (*v*. 4 % of those who were food sufficient), lack of a vehicle was not a significant predictor of food insufficiency in the fixed effects models (est. = 0·02, *P* = 0·38). The latter question asking about difficulty accessing food because they did not have a car was very strongly related to food insufficiency and was not included as a predictor of food insufficiency because of the high correlation and its formulation which makes the presence of food insufficiency more likely (this effect is denoted as N.S. in online supplementary material, Supplemental Table 2).

The receipt of some types of support and benefits was associated with food insufficiency risk, most with *lower* risk, but some with *higher* risk. The receipt of SNAP and Special Supplemental Nutrition Program for Women, Infants and Children was both associated with *reduced* food insufficiency, but this relationship was only statistically significant for SNAP (est. = –0·02, *P* = 0·02). Participants getting SNAP (*v*. not) were 22 % more likely to transition from food insufficiency to food sufficiency. Getting Pandemic EBT had a negative but non-significant association with food insufficiency (est. = –0·03, *P* = 0·097). Receipt of unemployment insurance, Social Security, Supplemental Security Income, economic stimulus funds and coronavirus aid was not significantly associated with a reduction in food insufficiency (see Fig. 3 and online supplementary material, Supplemental Table 2).

Having a greater proportion of one’s social circle who ‘provided help with food’ positively predicted food insufficiency (est. = 0·07, *P* = 0·03). This may reflect a process where people experiencing food insufficiency have social circles that *respond* to this need by providing help (rather than a process where receiving food help increases one’s risk for remaining food insufficient).

Joint tests for the effects of programmes and benefits received were also explored, because these explanatory variables are likely correlated (i.e. people who receive one type of benefit can be more likely to receive other benefits), and this correlation can lead to insignificant individual coefficients, while they are jointly significant. However, none of the joint tests was statistically significant. Finally, the model included coefficients of the *averages* of the time-varying variables, which represent the correlation of the unobserved individual characteristics with the time-varying explanatory variables (statistics available on request). We find that the individual effects that make one more likely to experience food insufficiency also make one significantly more likely to be unemployed and to receive SNAP, Social Security Disability Insurance and stimulus money.

## Discussion

The results of this study, which rapidly and regularly monitored food insufficiency in L.A. County, show that the COVID-19 pandemic quickly led to a rise in rates of food insufficiency among county residents. On average, 10 % of L.A. County residents (i.e. one million people) experienced past week food insufficiency between April and December 2020. This is aligned with national statistics reporting that the average rate of household food insecurity in California was 9·8 % between 2018 and December 2020^(8)^, based on surveys that assessed peoples’ experience of food insecurity over the previous 12 months. However, this annual survey masks the large fluctuation in rates captured by our *biweekly* surveys, which found that the highest rates of food insufficiency – up to 23 % past week food insufficiency – were observed at the onset of the pandemic. Historically, the county has monitored food insecurity only among low-income households (< 300 % FPL), and the proportion of low-income households that experienced food insecurity at some point *in the previous 12 months* was 31 % in 2011, 29 % in 2015 and 27 % in 2018^(45)^. In 2018, it is likely that approximately 5 % of low-income households experienced food insecurity in a given month^(45)^. Given the major shock of the pandemic to L.A. County’s economic, social and food systems, a spike in food insecurity was anticipated, but it was alarming to find that almost a quarter of the population experienced food insufficiency – the most severe dimension of food insecurity – within weeks of the pandemic onset.

Aligned with previous evidence^(12)^, this study found that poverty, low household income and less education were key risk factors for food insufficiency during the pandemic. Although race and ethnicity did not predict food insufficiency in our multivariate statistical models, Hispanic/Latinx residents of L.A. County were disproportionately impacted by food insufficiency. Thus, initiatives to provide food assistance to these populations that pre-dated the pandemic were likely well-positioned to reach communities at risk for food inequities when the pandemic hit. This study also found that residents with a larger household size had additional risk for food insufficiency. Because household size is accounted for when computing household poverty and low-income status, the independent effect of household size suggests that larger households, regarless of their income level, have an increased risk for food insufficiency, perhaps due to more food requirements.

One less expected finding was that adults aged 41–50 had the highest risk for food insufficiency during the first year of the pandemic, relative to other age groups. Historically younger adults are more likely to experience food insecurity and insufficiency than older adults^(12)^. But, at the onset of the pandemic, there was concern that low-income older adults (e.g. 65 and up) would face challenges getting enough food as they would be more likely to shelter at home, lose social and community supports, and because they may have less digital literacy to access emerging food assistance programmes. We found adults ≥ 65 years made up 17 % of our study population, but only 7 % of the sub-population that experienced food insufficiency, and in our statistical models they had the *lowest risk* for food insufficiency. Older adults appear to have been less impacted by the pandemic-related financial crisis, as they are less likely to be in the workforce (and thus impacted by unemployment) and more likely to have stable sources of incomes and savings. This age group may also have been well served by the programmes that emerged to deliver food to older adults^(46)^. However, low- to middle-income adults aged 41–50 were more likely to have lost jobs and incomes, and they may have dependents on ‘both sides’ (i.e. children and parents) and larger financial obligations, compared to their younger or older counterparts. This finding highlights the importance of quickly monitoring and identifying segments of the community experiencing food insecurity and insufficiency during a given crisis, to tailor the outreach and the response^(47)^.

The environments in which L.A. County residents live is varied, reflecting the diversity in cultural groups and landscapes that span the counties’ many cities – such as Compton, Los Angeles, Santa Monica and West Hollywood. Participants in our study who experienced food insufficiency had larger households, but smaller social networks, compared to those who were food sufficient. More than one in ten people in our sample (12 %) lived in a food desert, with poor access to a grocery store, and 21 % of people experiencing food insufficiency reported that they had difficulties getting food because they did not have a vehicle. These potential barriers to food access did not significantly increase Angelenos’ risk for food insufficiency. Nonetheless, they should be taken into consideration in future research as possible risk factors for broader experiences of food insecurity and in future efforts to make local food systems more fair and resilient^(5)^.

Finally, the results of this study provide evidence that governmental food assistance through the SNAP programme was associated with a lowered risk for food insufficiency. SNAP has historically ameliorated food insecurity^(11)^ and food insufficiency^(48)^ and appears to have continued to do so during the pandemic. The 20 % reduced risk for food insufficiency found in our results matches previous evidence: that SNAP programme participants are between 5 and 20 percentage points less likely to be food insecure than nonparticipants, after accounting for factors that lead people to enrol in the programme (including the experience of food insecurity itself)^(11)^. As food insufficiency spiked, SNAP’s reach into low-income communities expanded with the County government recording a 20 % increase in enrolments between March and August^(49)^. Although this is certainly a success, it is also worth noting that 15–27 % of L.A. County residents were likely eligible for SNAP, but *not* enrolled^(49)^. Historically, food insecure households may under-enrol in or not be eligible for SNAP^(50)^. It is also important to note that SNAP is not accessible for undocumented residents, and a limitation of our study is that we do not know if the sample is representative of the undocumented population, which is estimated to be 880 000 residents in L.A. County^(51)^.

Pandemic EBT was not significantly associated with food insufficiency. This newly established government food assistance programme provided additional assistance to families with children eligible for free or reduced-price school meals.^(34)^ Perhaps because its benefits were issued for children, receipt of pandemic EBT was not significantly associated with reduced food insufficiency for adult survey respondents. Other government benefits that provided financial assistance, including Social Security and the temporary economic stimulus funds and coronavirus aid, were not associated with lower food insufficiency risk, perhaps because this money was being spent on other pressing financial needs like delayed rent and other bills.

### Study strengths and limitations

A key strength is that the study participants constituted a large and representative sample of the L.A. County population, allowing us to infer population-level rates of food insufficiency, and risk factors and solutions for a large and diverse urban population. Additionally, the panel participants were surveyed every 2 weeks throughout the pandemic, providing longitudinal data with temporal richness in both the experience of food insufficiency and changes to participants’ use of food assistance supports, programmes and other benefits. This allowed us to test if change in these exposures predicted change in food insufficiency.

A limitation is that the food insufficiency measure used in this study, a subset of items from the FIES^(38)^, which was selected in part because of its’ brevity (an important criteria in large panel surveys), is not the same measure used historically by L.A. County to track food insecurity, the U.S.D.A.’s U.S. Adult Food Security Survey Module^(52)^. Also, for brevity, we used three of eight FIES items that are central as seen in item-total correlations^(38,39)^, but that focus on a more severe dimension of food insecurity (Table 1).

Because the study was not an experimental design, it is possible that the significant associations we observed in this longitudinal study (e.g. the receipt of SNAP being associated with a transition from food insufficiency to food sufficiency) may be explained by other unmeasured confounding variables. This limits our ability to identify causal effects. Also, because we considered brief 2-week periods between survey waves, the fixed effect model estimates reflected relatively short-term effects of time-varying variables (e.g. receipt of SNAP or stimulus funds) on food insufficiency, rather than potential longer-term effects (e.g. if the receipt of stimulus checks had an impact on food insufficiency over a longer timeframe).

A further limitation was that the U.S.D.A. measure of neighbourhood food access^(36)^ did not capture change in food outlets (closures, openings) related to the pandemic and associated mandates for business opening times and capacity.

## Conclusions

This study highlights the value of rapidly monitoring population food insecurity and insufficiency during a sustained crisis to determine the level of risk, *who* is at risk, and the barriers they face. We found that the COVID-19 pandemic caused high rates of food insufficiency in L.A. County, with poverty, low income, less education and larger households increasing food insufficiency risk. Middle aged adults (41–50 years old) were a segment of the population at especially heightened risk, who may require tailored outreach and interventions.

This study also finds evidence for the benefits of government food assistance programmes in alleviating food insufficiency during a crisis. Varied emergency food and financial assistance programmes were used by the many L.A. County residents who experienced food insufficiency in the first year of the COVID-19 pandemic, including help from family and friends, food pantries, and government emergency relief and sustained programmes. Although emergency food assistance can provide short-term benefits^(20)^, it was a sustained government food assistance programme – SNAP – that appears to have meaningfully alleviated food insufficiency. Efforts to quickly increase access to and enrolments in this type of government programme are likely to help reduce food insufficiency and insecurity in large urban populations during a sustained crisis.

## Supplementary material

For supplementary material accompanying this paper visit https://doi.org/10.1017/S1368980023001337. click here to view supplementary material

## References

[1] Bauer L (2020) Hungry at Thanksgiving: A Fall 2020 Update on Food Insecurity in the US. Brookings Institution. https://www.brookings.edu/articles/hungry-at-thanksgiving-a-fall-2020-update-on-food-insecurity-in-the-u-s/ (accessed December 2020).

[2] Wolfson JA & Leung CW (2020) Food Insecurity and COVID-19: disparities in early effects for US adults. Nutrients 12, 1648. 32498323 10.3390/nu12061648 PMC7352694

[3] Schanzenbach D & Pitts A (2020) How Much Has Food Insecurity Risen? Evidence from the Census Household Pulse Survey. Institute for Policy Research Rapid Research Report. https://www.ipr.northwestern.edu/documents/reports/ipr-rapid-research-reports-pulse-hh-data-10-june-2020.pdf (accessed July 2020).

[4] Center on Budget and Policy Priorities (2020) Tracking the COVID-19 Recession’s Effects on Food, Housing, and Employment Hardship. http://www.jstor.org/stable/resrep25613 (accessed August 2021).

[5] Hawkes C & Squires CG (2021) A double-duty food systems stimulus package to build back better nutrition from COVID-19. Nat Food 2, 212–214. 37118472 10.1038/s43016-021-00260-6

[6] U.S.D.A. (2019) Food Security Status of U.S. Households in 2018. https://www.ers.usda.gov/topics/food-nutrition-assistance/food-security-in-the-us/key-statistics-graphics.aspx (accessed August 2022).

[7] U.S.D.A. (2022) Food Security in the U.S.: Measurement. https://www.ers.usda.gov/topics/food-nutrition-assistance/food-security-in-the-u-s/measurement/#comparison (accessed August 8 2022).

[8] Coleman-Jensen A , Rabbitt MP , Gregory CA et al. (2021) Household Food Security in the United States in 2020, ERR-298. Washington, DC: U.S. Department of Agriculture, Economic Research Service.

[9] Livings MS , Bruine de Bruin W , Wilson JP et al. (2023) Food insecurity is under-reported in surveys that ask about the past year. Am J Prev Med, S0749-3797(23)00162-9 (online ahead of print). https://pubmed.ncbi.nlm.nih.gov/37028568/ 10.1016/j.amepre.2023.03.022 37028568

[10] Dubowitz T , Ghosh Dastidar M , Troxel WM et al. (2021) Food Insecurity in a low-income, predominantly African American cohort following the COVID-19 pandemic. Am J Public Health 111, 494–497. 33476228 10.2105/AJPH.2020.306041 PMC7893363

[11] Gundersen C & Ziliak JP (2018) Food insecurity research in the United States: where we have been and where we need to go. Appl Econ Perspect Policy 40, 119–135.

[12] Gundersen C & Ziliak JP (2015) Food insecurity and health outcomes. Health Aff 34, 1830–1839. 10.1377/hlthaff.2015.0645 26526240

[13] Carducci B , Keats EC , Ruel M et al. (2021) Food systems, diets and nutrition in the wake of COVID-19. Nat Food 2, 68–70. 37117413 10.1038/s43016-021-00233-9

[14] Center on Budget and Policy Priorities (2020) A Quick Guide to SNAP Eligibility and Benefits. https://www.cbpp.org/research/food-assistance/a-quick-guide-to-snap-eligibility-and-benefits (accessed November 2020).

[15] Hall, L & Nchako, C (2021) A Closer Look at Who Benefits from SNAP: State-by-State Fact Sheets. Center on Budget and Policy Priorities. https://www.cbpp.org/research/food-assistance/a-closer-look-at-who-benefits-from-snap-state-by-state-fact-sheets (accessed July 2021).

[16] Nord M & Golla AM (2009) Does SNAP decrease food insecurity? Untangling the self-selection effect. Economic Research Report No. 85, U.S. Dept. of Agriculture, Economic Research Service. https://www.ers.usda.gov/webdocs/publications/46295/10977_err85_1_.pdf?v=2544.7 (accessed September 2020).

[17] Swann CA (2017) Household history, SNAP participation, and food insecurity. Food Policy 73, 1–9.

[18] Feeding America (2014) Hunger in America Study. https://www.feedingamerica.org/research/hunger-in-america (accessed June 2021).

[19] RTI International (2014) Current and Prospective Scope of Hunger and Food Security in America: a Review of Current Research. https://www.rti.org/sites/default/files/resources/full_hunger_report_final_07-24-14.pdf (accessed June 2021).

[20] An R , Wang J , Liu J et al. (2019) A systematic review of food pantry-based interventions in the USA. Public Health Nutr 22, 1704–1716. 30834852 10.1017/S1368980019000144 PMC10260889

[21] Martin KS , Rogers BL , Cook JT et al. (2004) Social capital is associated with decreased risk of hunger. Social Sci Med 58, 2645–2654. 10.1016/j.socscimed.2003.09.026 15081212

[22] Cattell V (2001) Poor people, poor places, and poor health: the mediating role of social networks and social capital. Social Sci Med 52, 1501–1516. 10.1016/s0277-9536(00)00259-8 11314847

[23] Lauren BN , Silver ER , Faye AS et al. (2021) Predictors of households at risk for food insecurity in the United States during the COVID-19 pandemic. Public Health Nutr 24, 3929–3936. 33500018 10.1017/S1368980021000355 PMC8207551

[24] Santarossa S , Hill AB , Sitarik AR et al. (2021) Food insecurity in Detroit: exploring the relationship between patient-reported food insecurity and proximity to healthful grocery stores. Public Health Nutr 25, 954-963. 34325766 10.1017/S1368980021003128 PMC9991681

[25] Blumenberg E , Pinski M , Nhan LA et al. (2021) Regional differences in the impact of the COVID-19 pandemic on food sufficiency in California, April-July, 2020: implications for food programs and policies. Public Health Nutr 24, 3442-3450. 33928894 10.1017/S1368980021001889 PMC8144834

[26] Ratcliffe C , McKernan S-M & Zhang S (2011) How much does the supplemental nutrition assistance program reduce food insecurity? Am J Agric Econ 93, 1082–1098. 25197100 10.1093/ajae/aar026 PMC4154696

[27] Restrepo BJ , Rabbitt MP & Gregory CA (2021) The effect of unemployment on food spending and adequacy: evidence from coronavirus-induced firm closures. Appl Econ Perspect Policy 43, 185–204.

[28] Raifman J , Bor J & Venkataramani A (2021) Association between receipt of unemployment insurance and food insecurity among people who lost employment during the COVID-19 pandemic in the United States. JAMA Network Open 4, e2035884. 33512519 10.1001/jamanetworkopen.2020.35884 PMC7846943

[29] Stokols D (1992) Establishing and maintaining healthy environments: toward a social ecology of health promotion. Am Psychol 47, 6–22. 1539925 10.1037//0003-066x.47.1.6

[30] McLeroy KR , Bibeau D , Steckler A et al. (1988) An ecological perspective on health promotion programs. Health Educ Q 15, 351–377. 3068205 10.1177/109019818801500401

[31] Story M , Kaphingst KM , Robinson-O’Brien R et al. (2008) Creating healthy food and eating environments: policy and environmental approaches. Ann Rev Public Health 29, 253–272. 18031223 10.1146/annurev.publhealth.29.020907.090926

[32] Barnhill A , Palmer A , Weston CM et al. (2018) Grappling with complex food systems to reduce obesity: A U.S. public health challenge. Public Health Reports 133, 44S–53S. 10.1177/0033354918802793 PMC6243440 30426872

[33] FRED Economic Data (2020) Civilian Labor Force in Los Angeles County, CA. https://fred.stlouisfed.org/series/CALOSA7LFN (accessed April 2021).

[34] California Department of Social Services (2020) Pandemic EBT. https://www.cdss.ca.gov/home/pandemic-ebt (accessed April 2021).

[35] University of Southern California (2020) The Understanding America Study. https://uasdata.usc.edu/index.php (accessed June 2020).

[36] U.S.D.A. (2020) Food Environment Atlas. https://www.ers.usda.gov/data-products/food-environment-atlas/ (accessed April 2021).

[37] Angrisani M , Kapteyn A , Meijer E et al. (2019) Sampling and Weighting the Understanding America Study. CESR-Schaeffer Working Paper No. 004. 10.2139/ssrn.3502405 (accessed April 2021).

[38] Cafiero C , Viviani S & Nord M (2018) Food security measurement in a global context: the food insecurity experience scale. Measurement 116, 146–152.

[39] Cafiero C , Melgar-Quinonez HR , Ballard TJ et al. (2014) Validity and reliability of food security measures. Ann NY Acad Sci 1331, 230–248. 25407084 10.1111/nyas.12594

[40] McKechnie R , Turrell G , Giskes K et al. (2018) Single-item measure of food insecurity used in the National Health Survey may underestimate prevalence in Australia. Aust New Zealand J Public Health 42, 389–395. 30035843 10.1111/1753-6405.12812

[41] U.S. Department of Health & Human Services (2020) 2020 Poverty Guidelines. https://aspe.hhs.gov/2020-poverty-guidelines (accessed December 2020).

[42] Martinez JC , Clark JM & Gudzune KA (2019) Association of personal vehicle access with lifestyle habits and food insecurity among public housing residents. Prev Med Rep 13, 341–345. 30792950 10.1016/j.pmedr.2019.01.001 PMC6369228

[43] Mundlak Y (1978) On the pooling of time series and cross section data. Econometrica: J Econ Soc 46, 69–85.

[44] Los Angeles County Department of Public Social Services (2020) Department At-A-Glance. https://myapps.dpss.lacounty.gov/pls/apexprod/f?p=20200123002:10:109088835238048 (accessed April 2021).

[45] Los Angeles County Department of Public Health (2018) 2018 Los Angeles County Health Survey. http://publichealth.lacounty.gov/ha/hasurveyintro.htm (accessed May 2021).

[46] Workforce Development Aging & Community Services (2020) Great Plates. https://wdacs.lacounty.gov/greatplates/ (accessed June 2021).

[47] de la Haye K , Miller S , Weber K et al. (2020) The Impact of COVID-19 on Food Insecurity in Los Angeles County: April to May 2020. Los Angeles, CA: University of Southern California.

[48] Gundersen C , Kreider B & Pepper JV (2017) Partial identification methods for evaluating food assistance programs: a case study of the causal impact of SNAP on food insecurity. Am J Agric Econ 99, 875–893.

[49] de la Haye K , Miller S , Livings M et al. (2020) The Impact of COVID-19 on Food Insecurity in Los Angeles County: April to July 2020. Los Angeles, CA: University of Southern California.

[50] Gundersen C (2021) Viewpoint: a proposal to reconstruct the Supplemental Nutrition Assistance Program (SNAP) into a universal basic income program for food. Food Policy 101, 102096.

[51] Migration Policy Institute (2018) Profile of the Unauthorized Population: Los Angeles County, CA. https://www.migrationpolicy.org/data/unauthorized-immigrant-population/county/6037 (accessed April 2021).

[52] U.S.D.A. (2019) Six-Item Short Form of the Food Security Survey Module. https://www.ers.usda.gov/topics/food-nutrition-assistance/food-security-in-the-us/survey-tools.aspx#adult (accessed June 2020).

