# Supplementary material for: Ecological risk and protective factors for food insufficiency in Los Angeles County during the COVID-19 pandemic
Source: Public Health Nutr. 2023 Jul 5;26(10):1944–55. doi: 10.1017/S1368980023001337 (PMC10564600; doi:10.1017/S1368980023001337)
Supplement: Supplementary file 1 [file S1368980023001337sup001.docx]

**Supplemental Table 1.** Percent of the L.A. County adult population that experienced past week food insufficiency and received government benefits, by survey wave (weighted statistics)

| **Wave** | **Food insufficient** | | **SNAP** | | **WIC** | **UI** | **SS** | **SSI** | **SSDI** | **Stimulus funds** | **Coronavirus Aid** |
| --- | --- | --- | --- | --- | --- | --- | --- | --- | --- | --- | --- |
| April 1-14 | 23% | 12% | | 7% | | 4% | 16% | 6% | 3% | 2% | 2% |
| April 15-28 | 16% | 13% | | 8% | | 7% | 15% | 7% | 4% | 39% | 3% |
| April 29-May 12 | 12% | 13% | | 7% | | 9% | 16% | 6% | 4% | 47% | 4% |
| May 13-26 | 10% | 14% | | 6% | | 10% | 17% | 6% | 4% | 47% | 4% |
| May 27-June 9 | 11% | 15% | | 6% | | 11% | 16% | 6% | 4% | 40% | 4% |
| June 10-23 | 11% | 15% | | 7% | | 14% | 16% | 5% | 3% | 25% | 4% |
| June 24-July 7 | 9% | 16% | | 6% | | 15% | 16% | 6% | 3% | 18% | 2% |
| July 8-21 | 9% | . | | . | | 14% | . | . | . | . | . |
| July 22-Aug 4 | 8% | 13% | | 6% | | 14% | 16% | 5% | 3% | 12% | 2% |
| Aug 5-18 | 9% | 14% | | 7% | | 15% | 16% | 5% | 3% | 9% | 2% |
| Aug 19-Sep 1 | 9% | 12% | | 6% | | 13% | 17% | 5% | 2% | 7% | 1% |
| Sep 2-15 | 9% | 11% | | 6% | | 14% | 17% | 6% | 3% | 6% | 1% |
| Sep 16-29 | 8% | 12% | | 5% | | 13% | 17% | 6% | 3% | 6% | 1% |
| Sep 30-Oct 13 | 10% | 11% | | 6% | | 12% | 16% | 6% | 3% | 5% | 1% |
| Oct 14-27 | 7% | 14% | | 5% | | 12% | 16% | 5% | 3% | 5% | 2% |
| Oct 28-Nov 10 | 7% | 13% | | 6% | | 12% | 16% | 5% | 2% | 4% | 1% |
| Nov 11-24 | 8% | 12% | | 7% | | 12% | 16% | 4% | 3% | 5% | 1% |
| Nov 25-Dec 8 | 7% | 13% | | 6% | | 11% | 17% | 5% | 3% | 4% | 1% |
| Dec 9-Dec 22 | 8% | 13% | | 6% | | 10% | 17% | 6% | 3% | 4% | 1% |

SNAP = Supplemental Nutrition Assistance Program; WIC = Special Supplemental Nutrition Program for Women, Infants and Children; UI = Unemployment Insurance; SS = Social Security, SSI = Supplemental Security Income; SSDI = Social Security Disability Insurance.

*Note.* Most benefits were not assessed in UAS 252, July 8-21.

**Supplemental Table 2.** Fixed effects model results: Predictors of past week food insufficiency in L.A. County, April to December 2020

| **Explanatory variables** | **Estimate** | **p-value** | **Significance** |
| --- | --- | --- | --- |
| *Demographics and health* |  |  |  |
| Male | 0.01 | 0.43 |  |
| Age (referent: 18-30 years) |  |  |  |
| 31-40 years | -0.02 | 0.17 |  |
| 41-50 years | 0.04 | 0.03 | * |
| 51-64 years | -0.02 | 0.27 |  |
| 65+ years | -0.01 | 0.68 |  |
| Race and ethnicity (referent: Non-Hispanic White) |  |  |  |
| Hispanic/Latinx (White) | 0.00 | 0.84 |  |
| Black (non-Hispanic) | -0.03 | 0.22 |  |
| Asian (non-Hispanic) | -0.02 | 0.28 |  |
| All American Indian/Alaskan Native | -0.02 | 0.59 |  |
| All Hawaiian/Pacific Islander | -0.05 | 0.18 |  |
| All Others | 0.00 | 0.88 |  |
| Education (referent: GED or less) |  |  |  |
| Some college | -0.02 | 0.42 |  |
| College and above | -0.04 | 0.04 | * |
| Household income |  |  |  |
| Living in poverty (<100% FPL) | 0.05 | <0.001 | *** |
| Low-income (<300% FPL) | 0.02 | 0.06 |  |
| Unemployed | 0.02 | 0.16 |  |
| Unemployed: lagged one period | -0.02 | 0.17 |  |
| Not employed because of disability | N.S. |  |  |
| Diagnosed with coronavirus | 0.02 | 0.46 |  |
| *Household and Social Factors* |  |  |  |
| Household Size | 0.01 | 0.04 | * |
| Have children in the household | -0.01 | 0.49 |  |
| Single parent with children | 0.02 | 0.54 |  |
| Social circle size (log)^a^ | 0.00 | 0.99 |  |
| *Food Access and Environments* |  |  |  |
| Low food access neighborhood^b^ | -0.01 | 0.56 |  |
| Household does not have a vehicle^c^ | 0.02 | 0.38 |  |
| *Receipt of Support, Programs, and Benefits* |  |  |  |
| % of social circle that helped with food^d^ | 0.07 | 0.03 | * |
| SNAP | -0.02 | 0.02 | * |
| WIC | -0.02 | 0.42 |  |
| Pandemic EBT | -0.03 | 0.10 |  |
| Unemployed * unemployment insurance | -0.01 | 0.32 |  |
| Social Security (SS) | 0.02 | 0.54 |  |
| Supplemental Security Income (SSI) | -0.01 | 0.48 |  |
| Social Security Disability Insurance (SSDI) | 0.04 | 0.07 |  |
| Economic stimulus funds | 0.00 | 0.66 |  |
| Aid for people affected by the coronavirus | -0.02 | 0.15 |  |
| Constant | 0.12 | <0.01 | ** |
| Observations | 11413 |  |  |
| R-squared (within) | 0.012 |  |  |
| R-squared (overall) | 0.104 |  |  |
| R-squared (between) | 0.164 |  |  |

GED = Tests of General Educational Development; FPL = Federal Poverty Level; SNAP = Supplemental Nutrition Assistance Program; WIC = Special Supplemental Nutrition Program for Women, Infants, and Children; N.S. = not significant and not included in the final model

^a^Social circle size = the number of family and friends they have

^b^Low food access neighborhood = a census tract defined as a food desert based on the U.S.D.A.’s definition of “low access tract at 1 mile for urban areas or 10 miles for rural areas”

^c^Household does not have a vehicle = participant reported that their household does not own or lease any private vehicles

^d^% of social circle that helped with food = participant’s estimation of the proportion of their family and friends (social circle) that helped them to get enough food to eat, by sharing money, resources, or food

* *p*<0.05, ** *p*<0.01, *** p<.001
